# Ensemble based in transfer learning for cytological classification in pleural fluid

**DOI:** 10.3389/fdgth.2026.1849898

**Published:** 2026-06-10

**Authors:** Frida López-Córdova, Hugo Vega-Huerta, Gisella Luisa Elena Maquen-Niño, Jaime Cáceres-Pizarro, Ciro Rodriguez, David Calderón, Juan Gamarra-Moreno, Percy De-la-Cruz-VdV, Luis Guerra-Grados, Santiago Moquillaza-Henríquez, Oscar Benito-Pacheco, Ivan Adrianzén-Olano, Mario Chauca

**Affiliations:** 1 Universidad Nacional Mayor de San Marcos, Lima, Peru; 2 Universidad Nacional Pedro Ruiz Gallo, Lambayeque, Peru; 3 Hospital Nacional Cayetano Heredia, Lima, Peru; 4 Universidad Nacional Toribio Rodríguez de Mendoza de Amazonas, Bagua, Peru; 5 Universidad Ricardo Palma, Lima, Peru

**Keywords:** cytology, deep learning, pleural fluid, transfer learning, voting classifier

## Abstract

Pleural effusion cytology is critical for diagnosing benign and malignant conditions, yet manual interpretation remains time-consuming and prone to subjectivity. The increasing burden of malignant pleural effusion in resource-constrained settings highlights the need for automated diagnostic solutions. This study presents an ensemble deep learning framework combining ResNet50V2, DenseNet121, and InceptionV3 architectures with transfer learning for classifying pleural cytology images into negative for malignancy (NFM) and malignant (MAL) categories. Three scenarios were evaluated: no data augmentation, 50% augmentation, and 300% augmentation. A local dataset of 1,292 images from Hospital Nacional Cayetano Heredia and an external Kaggle dataset (693 images) were used for training, validation, and independent testing. Performance was measured using accuracy, precision, recall, and *F*1-score across individual models and ensemble voting strategies (hard and soft voting). The ResNet + DenseNet ensemble with soft voting and 300% data augmentation achieved the highest accuracy (96.2% on the local dataset; 89.6% on the external dataset), outperforming individual models across all scenarios. Increasing the dataset through data augmentation significantly improved generalization and robustness. The proposed ensemble-based approach supports cytological diagnosis, potentially reducing diagnostic uncertainty in pleural carcinoma detection. Our findings demonstrate that ensemble deep learning models, optimized with data augmentation, can provide accurate and reproducible diagnostic support for pleural cytology, offering practical potential for deployment in low-resource healthcare settings and contributing to improved cancer diagnosis accessibility.

## 1 Introduction

Pleural effusion (PE) is a pathological condition characterized by an excessive accumulation of fluid in the pleural space. PE is commonly associated with systemic disorders and organ dysfunction (1), and can occur secondary to different neoplasms, including lung, breast, and ovarian cancers (2).

More than 1.5 million cases are diagnosed annually in the United States, with an estimated overall incidence of approximately 360 cases per 100,000 inhabitants. PE can be associated with benign etiologies, such as congestive heart failure, or malignant ones, such as lung and breast cancer, representing an important marker of morbidity and mortality (3).

In developing countries, infectious etiologies—particularly tuberculosis—account for a substantial proportion of PE cases. An estimated 20%–25% of pleural effusions in these settings are attributable to this disease. In Peru, where the tuberculosis incidence reaches approximately 116 cases per 100,000 inhabitants, PE as an extrapulmonary manifestation carries significant clinical relevance. Moreover, malignant pleural effusions, associated with advanced cancers, represent a growing clinical burden due to increasing life expectancy and the epidemiological transition towards chronic non-communicable diseases (4).

Early detection and accurate classification of cancer cells are critical for improving patient outcomes. However, the identification of malignant cells in cytological preparations remains challenging due to morphological heterogeneity and inter-observer variability (5).

Approximately 50% of PEs are neoplastic, requiring cytological examination of pleural fluid for diagnostic confirmation. Cytological analysis is essential to confirm malignancy, identify cancerous and pre-cancerous cells, and detect other pathological conditions (6).

The cytological evaluation of serous effusions relies on the morphological and textural identification of cells within the fluid—a labor-intensive process subject to significant inter-observer variability (7).

Malignant pleural effusion (MPE) is defined as the confirmed presence of malignant cells in pleural fluid following cytological examination (8).

Cytology is a widely used and effective technique for the diagnosis of MPE, which is perceived to be easy, cost-effective, and quick (5). Early test results contribute to optimal care, access to treatment, and patient improvement. If there is clinical suspicion of MPE with negative cytology, further tests should be performed. However, additional tests are expensive, time-consuming, and sometimes very invasive for the patient. In this context, and due to the limitations of the cytological study, including the subjective nature of the specialist and the possibility of human error in diagnosis, many researchers have been exploring diagnostic techniques in gynaecology and radiology imaging. Moreover, the incursion into pathology is a growing field of research with new challenges that could be observed in the literature review, where studies were found to diagnose different types of neoplasms. However, to diagnose cancer cells in pleural effusion, much work remains to be done (9).

In this context, artificial intelligence (AI), particularly deep learning (DL), has emerged as a promising approach to overcome the limitations of conventional cytological diagnosis (10–12). Recent advances in convolutional neural networks (CNNs) have demonstrated strong performance in medical image classification tasks, including cancer detection from histopathological and cytological preparations (13, 14).

In conventional practice, a pathologist examines processed pleural fluid slides under an optical microscope, visually analyzing cellular morphology to reach a diagnosis. This process is inherently time-consuming and subject to inter-observer variability, compounded by a shortage of specialized pathologists, particularly in developing countries such as Peru.

Transfer learning enables the adaptation of CNN architectures pre-trained on large-scale datasets to domain-specific tasks, which is particularly advantageous when labeled medical data are scarce (15). In this study, three complementary architectures were selected: ResNet50V2, DenseNet121, and InceptionV3, each offering distinct feature extraction capabilities suited to medical image classification. The technical details of these architectures are described in Section 3.3.

Accordingly, this study addresses the following research question: Can ensemble models based on transfer learning architectures improve the accuracy and robustness of cytological classification of pleural fluid compared to individual CNN models? The objective is to design and evaluate ensemble deep learning models, combining ResNet50V2, DenseNet121, and InceptionV3, through voting strategies—for the automatic classification of pleural cytology images into two diagnostic categories (malignant and negative for malignancy), following the criteria of the International System for Reporting Serous Fluid Cytopathology (TIS). The proposed system is validated on a local clinical dataset and benchmarked against an external dataset to assess generalizability. The authors acknowledge that TIS comprises five diagnostic categories (ND, NFM, AUS, SFM, and MAL). However, this study restricted analysis to NFM and MAL for three reasons: first, intermediate categories (AUS, SFM) exhibit markedly low frequency in multicenter series, collectively under 3%, making statistically representative institutional sampling unfeasible (16, 17); second, NFM and MAL together represent over 90% of serous effusion specimens, constituting the diagnostic core of TIS (17, 18); and third, binary classification is a widely accepted strategy in early-stage AI cytopathology model development (19). Future work should extend the model to all five categories.

The main contributions of this work are: (1) the systematic combination of three complementary CNN architectures with ensemble voting to exploit their individual strengths; (2) a demonstrated improvement in classification performance over individual models on pleural cytology images; (3) validation across both local and external datasets, providing evidence of cross-domain generalizability; and (4) a clinically oriented workflow designed as a decision-support tool for pathologists rather than a fully autonomous diagnostic system.

## 2 Related works

The application of deep learning and machine learning techniques to cytological and histopathological image analysis has gained substantial momentum in recent years, with numerous studies demonstrating their potential to improve diagnostic accuracy across diverse clinical domains. Table 1 summarizes the key studies reviewed in this section, including the models employed, datasets used, performance metrics, and reported values.

**Table 1:** Summary of related works.

Citation	ML model	Dataset	Performance metrics	Values
Thakur et al. (13)	CNN (Systematic Review of 26 studies)	Non-gynecological cancer cytopathology (thyroid, bladder, lung, breast, pleural, ovary, pancreas, prostate)	Accuracy (reported across reviewed studies)	Close to 100% (thyroid PTCA on 908 WSIs); 100% sensitivity and specificity (breast)
Slabaugh et al. (11)	ML/DL (Literature Review)	Thyroid FNA cytology and histopathology	FNA sensitivity; FNA specificity (reported range)	68%–98%; 56%–100%
Yu et al. (20)	CNN (two models)	Cerebrospinal fluid cytology (leptomeningeal metastasis)	Accuracy (cell type); mAP (cancer subtype)	96.15%; 78%
Aboobacker et al. (21)	U-Net	Pleural fluid cytology images	Precision; Recall; Specificity; AUC-ROC	0.96; 0.96; 0.97; 0.97
Xie et al. (14)	Deep CNN (DCNN)	Cytological pleural effusion images (404 cases; 60 test WSIs)	Accuracy; Sensitivity; Specificity; AUC-ROC	91.67%; 87.50%; 94.44%; 0.9526
Wang et al. (22)	Interpretable Multi-scale Attention with Self-Supervised Learning (IMA-SSL)	Pleural effusion cytology WSIs (cell blocks and smears; 1,250 WSIs)	AUROC; MeanSS; Accuracy	Outperformed 5 SOTA models across 3 tasks (*p* < 0.001); specific numerical values reported within the study for 1,250 WSIs
Carlo Bruno et al. (23)	PatchCore (ResNet-50 backbone; anomaly detection)	Cytology images (image-level annotation)	Recall; Precision; AUROC (best embedding-based model)	1.00; 98.39%; 99.98%
Madathil et al. (24)	Multimodal DL (DenseNet121 + Transformer-based fusion)	Cervical clinical + colposcopic data (6,356 cases)	Accuracy (clinical only); AUC (clinical only); Accuracy (multimodal); AUC (multimodal)	82%; 88%; 90%; 95%
De Oliveira et al. (25)	Hybrid (Transfer Learning + Ensemble classifier)	Histological images	Accuracy (breast cancer); Accuracy (best hybrid model)	98.00%; 99.32% (ResNet-50 deep features + ensemble classifier on UCSB and LG datasets of breast, colorectal and liver tissue)

In the broader context of cancer cytopathology, a systematic review of 26 AI studies applied to non-gynecological cancers, encompassing organs such as the thyroid, bladder, lung, breast, pleural cavity, ovary, pancreas, and prostate, revealed that deep learning models achieved near-perfect accuracy for papillary thyroid carcinoma classification on 908 whole-slide images, along with 100% sensitivity and specificity for breast cytopathology (13). Similarly, a comprehensive review of machine learning and deep learning applications for thyroid cytology and histopathology reported that fine-needle aspiration cytology sensitivity ranged from 68% to 98% and specificity from 56% to 100%, while emphasizing the need for interpretability, cross-laboratory robustness, and domain adaptation strategies such as federated learning (11).

For cerebrospinal fluid cytology, two CNN models were developed to classify cancer cells in leptomeningeal metastasis, achieving 96.15% accuracy for cell type identification and a mean average precision of 78% for cancer subtype classification, along with a computer-aided diagnosis system that reduced analysis time by 90% (20).

The literature reflects a growing body of evidence supporting ensemble architectures in cytopathological image analysis. In serous effusion cytology specifically, Abd-Almoniem et al. (26) constructed a TIS-aligned real-world dataset of 3,731 images, demonstrating that VGG16 combined with transfer learning and random forest classification is a viable approach for multiclass cytological diagnosis, establishing an important methodological precedent for AI in effusion cytology. More broadly, Hossain et al. (27) demonstrated, via a max voting ensemble of MobileNetV2, VGG16, ResNet50, DenseNet121, InceptionV3, and Xception, that individual model accuracies of 77.2%–91.9% can be systematically exceeded by combining complementary architectural features. These findings from adjacent oncological imaging domains collectively validate the selection and ensemble combination of CNN architectures adopted in the present study and reinforce the broader applicability of voting-based ensemble strategies in transfer learning frameworks for medical image classification.

Regarding pleural effusion cytology, the primary focus of the present study, several contributions have demonstrated the viability of deep learning for this task. A U-Net architecture was employed to detect malignant cells in pleural fluid cytology images, achieving a precision of 0.96, recall of 0.96, specificity of 0.97, and an AUC-ROC of 0.97 (21). In another study, a deep CNN was applied to classify negative for malignancy and malignant cells on 404 cytological pleural effusion cases, reporting 91.67% accuracy, 87.50% sensitivity, 94.44% specificity, and an AUC-ROC of 0.9526 on 60 test whole-slide images (14).

More recently, an interpretable multi-scale attention framework with a self-supervised learning encoder (IMA-SSL) was proposed for pleural effusion cytology, which consistently outperformed five state-of-the-art models across three diagnostic tasks on a dataset of 1,250 whole-slide images, with statistical significance confirmed by Fisher's exact test (*p* < 0.001) (22).

Beyond pleural cytology, recent advances in label-efficient and multimodal methodologies offer relevant insights. A weakly supervised anomaly detection approach using the PatchCore algorithm with a ResNet-50 backbone achieved perfect recall (1.00), 98.39% precision, and an AUROC of 99.98% on cytology images using only image-level annotations demonstrating the feasibility of reducing annotation burden without sacrificing diagnostic performance (23). In the domain of cervical cancer screening, a multimodal deep learning model combining clinical history with colposcopic images via a Transformer-based fusion strategy achieved 82% accuracy and 88% AUC using clinical data alone, which improved to 90% accuracy and 95% AUC when colposcopy images were integrated, highlighting the benefit of multimodal data fusion (24).

Finally, hybrid approaches combining transfer learning features with ensemble classifiers have demonstrated strong classification performance on histological images. Using deep features from ResNet-50 with an ensemble of five algorithms, the best hybrid models achieved 98.00% and 99.32% accuracy on breast, colorectal, and liver tissue datasets, supporting the rationale for ensemble-based classification strategies in cytopathological analysis (25).

Collectively, these studies underscore the growing effectiveness of CNN-based architectures, transfer learning, and ensemble methods in medical image classification. However, their application to pleural effusion cytology remains limited compared to other domains. As evidenced in the reviewed literature, ensemble approaches have been successfully applied to other cytological and histopathological domains (25, 28), and individual deep learning models have been used for pleural effusion analysis (14, 21, 22). Nevertheless, to the best of the authors' knowledge, no prior study has combined multiple pre-trained CNN architectures with ensemble voting strategies specifically for pleural fluid cytology classification, a gap that the present work aims to address.

## 3 Methods

All experiments were conducted using Jupyter Notebook as the interactive development environment, with Python as the programming language and TensorFlow, Keras, and OpenCV as the primary libraries for model development, image processing, and evaluation.

**Figure 1:**
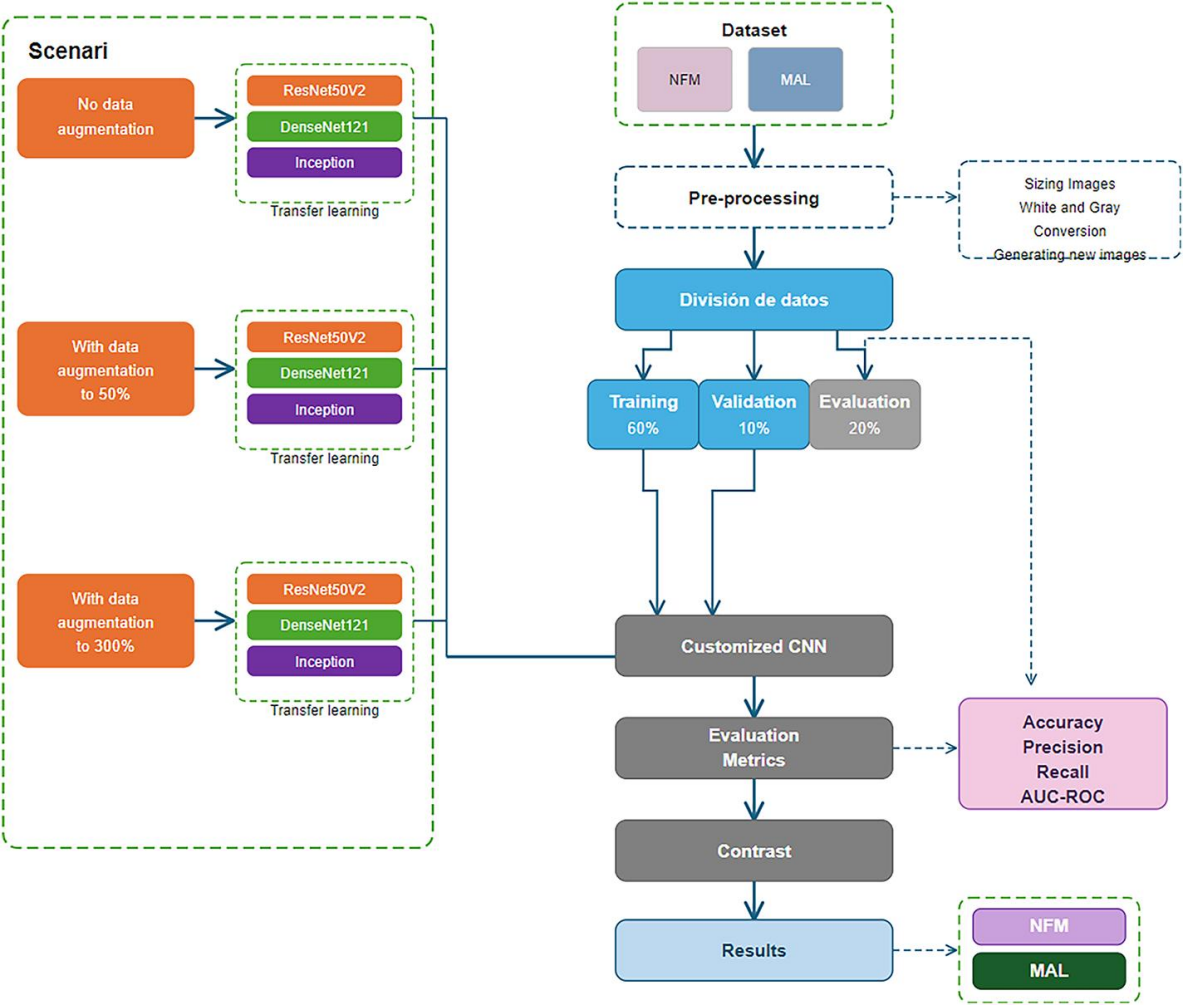
Research methodology.

The overall methodology, illustrated in Figure 1, comprises four main stages: (1) dataset construction from clinical pleural fluid cytology images; (2) image preprocessing, including resizing, grayscale conversion, and data augmentation; (3) model training using three transfer learning architectures combined with custom classification layers; and (4) performance evaluation through individual and ensemble voting classifiers. The dataset was partitioned into 60% for training, 10% for validation, and 30% for testing, maintaining class balance across all subsets.

Three data augmentation scenarios were designed to assess the impact of training set size on classification performance: Stage 1 without augmentation (775 training images), Stage 2 with 50% augmentation (1,163 training images), and Stage 3 with 300% augmentation (3,101 training images). In all three stages, the validation (129 images) and test (388 images) sets remained identical and composed exclusively of original, unaugmented images. All images were classified into two diagnostic categories: malignant (MAL) and negative for malignancy (NFM), following the criteria of the International System for Reporting Serous Fluid Cytopathology (TIS).

An empirical sensitivity analysis was incorporated comparing different levels of data magnification: 0%, 50%, 100%, 200% and 300%. The value of 300% was adopted as the upper limit because it allowed the performance of the minority classes to be improved without showing overfitting or excessive generation of artificially redundant images. The final selection was based on the performance obtained from each increase in the validation set, with the percentages being 50% and 300% as those selected. The entire augmentation process was applied exclusively to the training set, avoiding information leakage to validation or testing.

### 3.1 Datasets

The dataset was constructed in collaboration with the Department of Anatomic Pathology of the Hospital Nacional Cayetano Heredia (HNCH), Lima, Peru, following ethical approval granted by the institution's Ethics Committee (authorization code 102-2024, dated 12/12/2024). Pleural fluid samples were processed according to standard cytological protocols: specimens were centrifuged, and the resulting cell pellets were transferred onto glass slides. The work consisted of studying sheets of pleural fluid smears previously colored with standard technique for pap smears, work carried out by the pathological anatomy service of the Cayetano Heredia Hospital (29). Microscopic images were acquired using an Olympus optical microscope equipped with an integrated Olympus DP72 digital camera, with a 10× eyepiece and a 60× objective lens, resulting in a total magnification of 600×. Images were captured at different magnifications (10× and 60×) to allow for both cell localization and detailed cellular analysis. All images were saved in PNG format at a native resolution of 4,140 × 3,096 pixels to preserve visual fidelity without compression artifacts, with file sizes ranging between 11 and 12 MB (29). A total of 1,292 cytological images were selected from the larger dataset and subsequently resized to 250 × 250 pixels during preprocessing. Two specialist pathologists with 20 and 8 years of experience, respectively, independently reviewed and annotated each image into the corresponding diagnostic category: 645 images classified as malignant (MAL) and 647 as negative for malignancy (NFM) (29). The diagnostic categories and image distribution are presented in Table 2.

**Table 2:** Diagnostic category and number of images in the HNCH dataset.

Category	Description	Images
MAL	Malignant	645
NFM	Negative for malignancy	647
TOTAL	Total	1,292

### 3.2 Image pre-processing

Image preprocessing involved a series of transformations applied to the cytological images to improve their representation in the feature space and facilitate the learning process (30).

**Figure 2:**
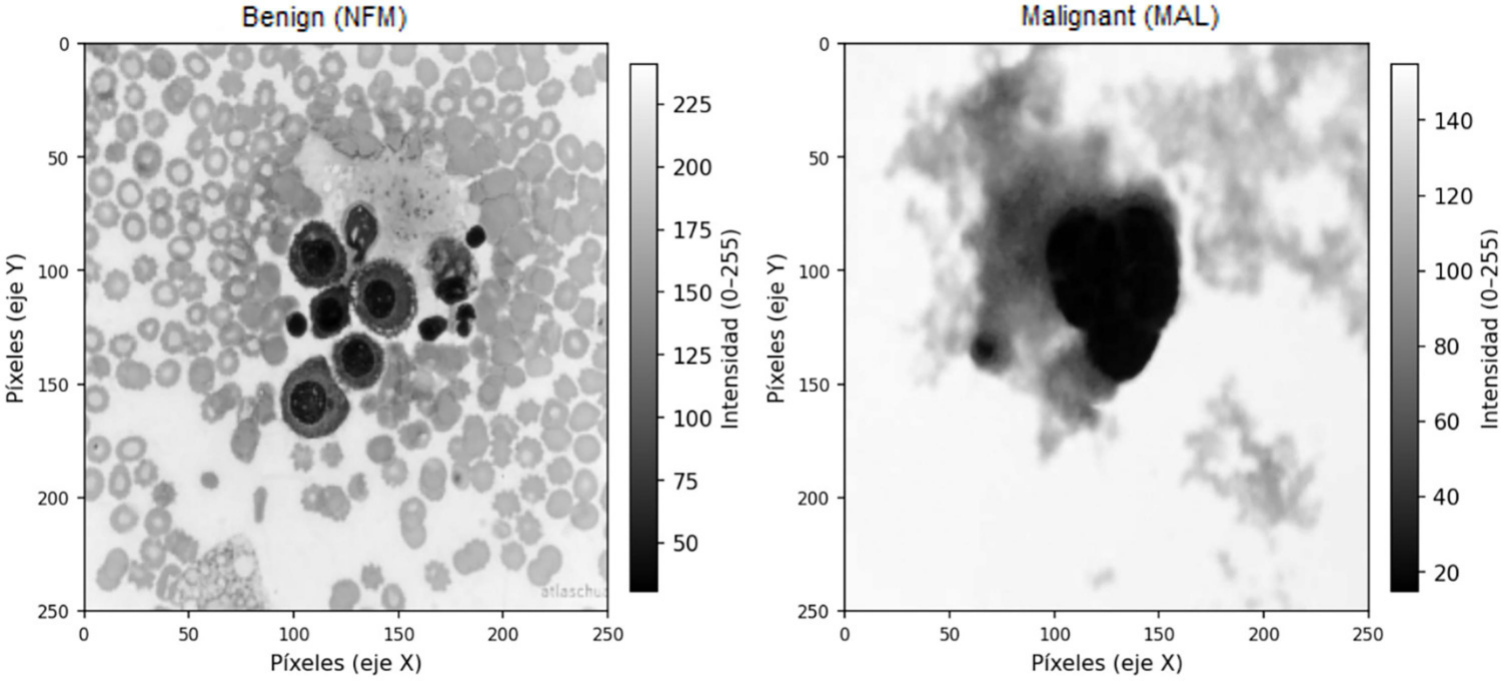
Grayscale two images of the MAL and NFM categories.

First, the images in the dataset were resized to 250 × 250 pixels using the Python RESIZE function. Next, the images were converted to grayscales using the IMREAD_GRAYSCALE function, since the original samples were obtained with different stains, resulting in background color variations that did not contribute to model learning. Figure 2 shows two images of the MAL and NFM categories in the greyscale.

**Figure 3:**
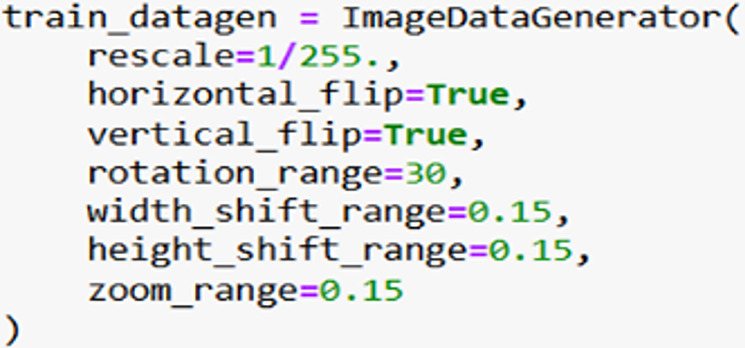
Image data generator parameters for image transformation.

The final preprocessing step involved data augmentation, a regularization technique that helps reduce overfitting by artificially expanding the training dataset through geometric transformations applied to the original cytological images in real time. For this purpose, the Keras ImageDataGenerator function was used with the parameters shown in Figure 3.

Pixel values were normalized by dividing each value by 255 (rescale = 1/255), transforming the original intensity range from [0, 255] to [0, 1] in order to accelerate convergence, stabilize training, and improve overall model performance. Several geometric transformations were then configured to augment the training data. Random horizontal and vertical flips, each with a 50% probability (horizontal_flip = True, vertical_flip = True), were enabled to generate mirror-image and vertically inverted versions of the cytological images, increasing the model's invariance to reflection and orientation changes. A random rotation of up to ±30 degrees (rotation_range = 30) was applied to simulate natural variations in object orientation and improve rotational invariance. Translation transformations were also introduced through random horizontal and vertical shifts of up to 15% of the image dimensions (width_shift_range = 0.15, height_shift_range = 0.15), reducing the model's dependence on specific spatial positions and improving generalization for off-center objects. Finally, a random zoom with a range of 15% (zoom_range = 0.15) was configured to simulate variations in capture distance, enhancing the model's ability to recognize objects at different scales.

Parameter values were set conservatively to preserve the semantic integrity of the cytological images while maximizing training diversity. These transformations—horizontal flip, vertical flip, rotation, width shift, height shift, and zoom—were applied exclusively to the training subset after the dataset had been partitioned into training, validation, and test sets. This ensures that no augmented variant of an original image used for training could appear in the validation or test sets, thereby preventing data leakage. The generated images were saved and merged only with the training partition to form the final augmented training set, while the validation and test sets remained composed entirely of original, unaugmented images.

### 3.3 Model architecture for image classification

Following feature extraction, the dataset was split into training (60%), validation (10%), and testing (30%) subsets. Based on the findings of a prior study (31), a hybrid architecture was implemented that combines pre-trained convolutional models as feature extractors with custom classification layers. Three base architectures were selected: ResNet50V2, DenseNet121, and InceptionV3, all pre-trained on ImageNet. These architectures were chosen for their complementary feature extraction capabilities: ResNet50V2 employs residual connections to mitigate gradient degradation in deep networks, DenseNet121 uses dense connectivity to promote feature reuse and reduce parameter redundancy, and InceptionV3 captures multi-scale features through parallel convolutions of varying kernel sizes.

For each base architecture, the convolutional layers were frozen to retain their pre-trained weights, and a custom classification head was appended with the following sequential structure: a global average pooling layer to reduce spatial dimensions, a dense layer with 512 units, a dropout layer for regularization, a Gaussian noise layer to improve generalization, a second dense layer with 128 units, a second dropout layer, and a final dense output layer with 2 units and softmax activation for binary classification. Table 3 presents the detailed layer configuration for each architecture.

**Table 3:** CNN architecture with a base architecture.

Base architecture	Layer (type)	Output shape
ResNet50v2	resnet50v2 Base architecture	(None, 8, 8, 2,048)
	global_average_pooling2d_1 (GlobalAveragePooling2D)	(None, 2,048)
	dense_1 (Dense)	(None, 512)
	dropout_1 (Dropout)	(None, 512)
	gaussian_noise (GaussianNoise)	(None, 512)
	dense_2 (Dense)	(None, 128)
	dropout_2 (Dropout)	(None, 128)
	dense_3 (Dense)	(None, 2)
DenseNet121	densenet121 Base architecture	(None, 7, 7, 1,024)
	global_average_pooling2d_2 (GlobalAveragePooling2D)	(None, 1,024)
	dense_1 (Dense)	(None, 512)
	dropout_1 (Dropout)	(None, 512)
	gaussian_noise (GaussianNoise)	(None, 512)
	dense_2 (Dense)	(None, 128)
	dropout_2 (Dropout)	(None, 128)
	dense_3 (Dense)	(None, 2)
Inception_v3	inception_v3 Base architecture	(None, 6, 6, 2,048)
	global_average_pooling2d_3 (GlobalAveragePooling2D)	(None, 2,048)
	dense_1 (Dense)	(None, 512)
	dropout_1 (Dropout)	(None, 512)
	gaussian_noise (GaussianNoise)	(None, 512)
	dense_2 (Dense)	(None, 128)
	dropout_2 (Dropout)	(None, 128)
	dense_3 (Dense)	(None, 2)

To further improve classification performance, an ensemble voting classifier was implemented to combine the predictions of the three individual architectures. Two aggregation strategies were explored: hard voting, in which the final prediction corresponds to the majority class among the individual model outputs, and soft voting, in which the predicted class probabilities are averaged across models and the class with the highest mean probability is selected. The following ensemble combinations were evaluated: ResNet50V2 + DenseNet121, ResNet50V2 + InceptionV3, DenseNet121 + InceptionV3, and ResNet50V2 + DenseNet121 + InceptionV3.

### 3.4 Distribution of the datasets

The original dataset of 1,292 images was first partitioned into three subsets following established machine learning protocols: 60% for training (775 images), 10% for validation (129 images), and 30% for testing (388 images). Stratified random sampling preserved the original class distribution across all subsets to ensure unbiased evaluation. Prior to partitioning, all images underwent quality assessment to exclude corrupted samples. Data augmentation was then applied exclusively to the training subset, ensuring strict separation between the augmented training data and the unmodified validation and test sets. This partitioning protocol prevents data leakage, a methodological risk that arises when augmented versions of training images inadvertently appear in the evaluation sets, leading to artificially inflated performance metrics (32). Three augmentation stages were defined to evaluate the effect of training set size on model performance, as described below.

#### 3.4.1 Stage 1: without data augmentation

The original 1,292 images were used directly, as shown in Table 4.

**Table 4:** Distribution of images without data augmentation (Stage 1).

Category	Initial	Train (60%)	Val (10%)	Test (30%)
MAL	645	387	65	194
NFM	647	388	65	194
TOTAL	1,292	775	129	388

#### 3.4.2 Stage 2: generation of new images at 50%

Data augmentation was applied to the training subset, generating 388 additional images to expand the training set from 775 to 1,163 images, as shown in Table 5.

**Table 5:** Distribution of images generated at 50% augmentation (Stage 2).

Category	Initial	Train (60%)	Augmented (50% of train)	Train augmented	Val (10%)	Test (30%)
MAL	645	387	194	581	65	194
NFM	647	388	194	582	65	194
TOTAL	1,292	775	388	1,163	129	388

#### 3.4.3 Stage 3: generation of new images at 300%.

A more aggressive augmentation factor was applied to the training subset, generating 2,326 additional images to expand the training set from 775 to 3,101 images, as shown in Table 6.

**Table 6:** Distribution of images generated at 300% augmentation (Stage 3).

Category	Initial	Train (60%)	Augmented (300% of train)	Train augmented	Val (10%)	Test (30%)
MAL	645	387	1,161	1,548	65	194
NFM	647	388	1,165	1,553	65	194
TOTAL	1,292	775	2,326	3,101	129	388

## 4 Results

### 4.1 Training and validation results of each CNN

This section presents the training and validation outcomes for each CNN architecture across the three data augmentation stages. All models were trained with early stopping and adaptive learning rate scheduling to optimize convergence.

#### 4.1.1 Stage 1: without data augmentation

In Stage 1, the original dataset (1,292 images) was used without augmentation. Table 7 presents the training parameters and results for each architecture.

**Table 7:** Parameters and results obtained during training and validation (Stage 1).

Category	Feature	ResNet	DenseNet	Inception
Parameters	Epochs	82/100	100/100	77/100
	# Steps	30	20	20
	Time × step	4/70 s	3/57 s	2/32 s
	Learning rate	2.00 × 10^−5^	1.00 × 10^−4^	2.00 × 10^−5^
Results	Loss	0.1167	0.2316	0.1054
	Accuracy	0.9561	0.9058	0.9626
	Val_loss	0.1434	0.1631	0.128
	Val_accuracy	0.9302	0.9302	0.9535

InceptionV3 achieved the highest training accuracy (0.9626) and validation accuracy (0.9535), with the lowest loss and validation loss among the three architectures, indicating an excellent fit with minimal overfitting. ResNet50V2 obtained a training accuracy of 0.9561 and a validation accuracy of 0.9302, demonstrating good generalization with low error in both stages. DenseNet121 recorded the lowest training accuracy (0.9058); however, its validation accuracy (0.9302) matched that of ResNet50V2, suggesting strong generalization despite a lower fit to the training data.

**Figure 4:**
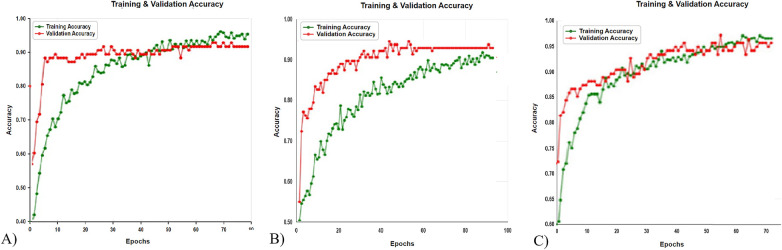
Training and validation results in Stage 1 for **(A)** ResNet50V2, **(B)** DenseNet121, **(C)** inception.

Figure 4 shows the accuracy and loss curves during training and validation for Stage 1. The green line corresponds to the precision and the red line to the loss value of the (A) ResNet50V2, (B) DenseNet121, (C) Inception model in Stage 1.

#### 4.1.2 Stage 2: generation of new images at 50%

In Stage 2, the training set was expanded to 1,163 images through 50% augmentation. Table 8 presents the corresponding training parameters and results.

**Table 8:** Parameters and results obtained during training and validation (Stage 2).

Category	Feature	ResNet	DenseNet	Inception
Parameters	Epochs	55/100	89/100	51/100
	# Steps	30	30	30
	Time × step	3/83 s	3/90 s	2/51 s
	Learning rate	2.00 × 10^−5^	4.00 × 10^−6^	2.00 × 10^−5^
Results	Loss	0.1785	0.3039	0.1765
	Accuracy	0.9296	0.8625	0.939
	Val_loss	0.2605	0.2512	0.2852
	Val_accuracy	0.8	0.9175	0.8711

InceptionV3 achieved the highest training accuracy (0.9390), followed by ResNet50V2 (0.9296) and DenseNet121 (0.8625). However, validation accuracy revealed a different pattern: DenseNet121 attained the highest validation accuracy (0.9175), while ResNet50V2 dropped to 0.8000 and InceptionV3 reached 0.8711. The gap between training and validation accuracy in ResNet50V2 (0.1296) and InceptionV3 (0.0679) suggests a tendency toward overfitting at this augmentation level, whereas DenseNet121 showed improved generalization with validation accuracy exceeding training accuracy by 0.0550. This behavior may be attributed to DenseNet's dense connectivity, which promotes feature reuse and regularization, making it more robust to moderate augmentation (33).

**Figure 5:**
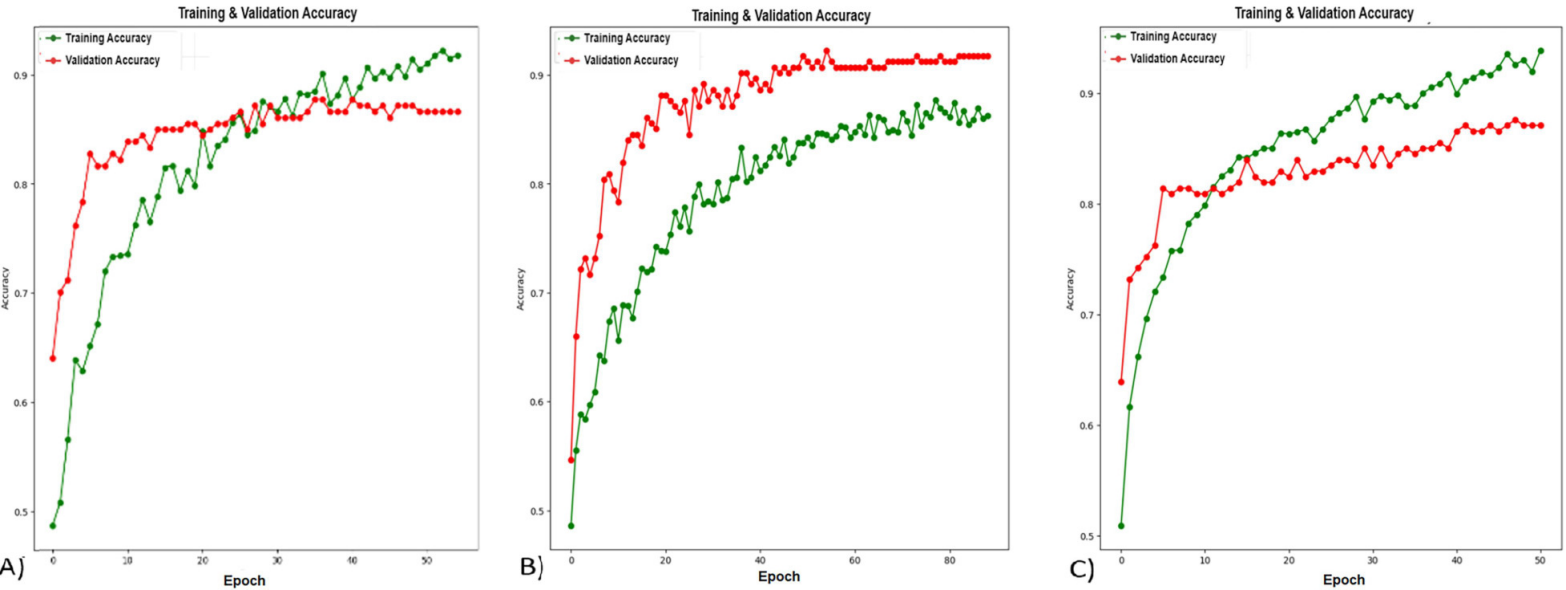
Training and validation results in Stage 2 for **(A)** ResNet50V2, **(B)** DenseNet121, **(C)** inception.

Figure 5 shows the graphs of accuracy and loss curve during training and validation for Stage 2. The green line represents precision, and the red line represents the loss value of the (A) ResNet50V2, (B) DenseNet121, (C) Inception model in Stage 2.

#### 4.1.3 Stage 3: generation of new images at 300%

In Stage 3, the training set was expanded to 3,101 images through 300% augmentation. Table 9 presents the training parameters and results.

**Table 9:** Parameters and results obtained during training and validation (Stage 3).

Category	Feature	ResNet	DenseNet	Inception
Parameters	Epochs	52/100	100/100	68/100
	# steps	78	78	78
	Time × step	2/182 s	3/227 s	2/129 s
	Learning rate	4.00 × 10^−6^	2.00 × 10^−5^	4.00 × 10^−6^
Results	Loss	0.1015	0.1412	0.1013
	Accuracy	0.9584	0.9423	0.9635
	Val_loss	0.1681	0.1884	0.1668
	Val_accuracy	0.9323	0.9188	0.9284

With the largest augmented dataset, all three architectures exhibited improved and more balanced performance compared to Stage 2. InceptionV3 achieved the highest training accuracy (0.9635) and the lowest training loss (0.1013), while ResNet50V2 attained the highest validation accuracy (0.9323). DenseNet121 recorded a training accuracy of 0.9423 and a validation accuracy of 0.9188. The reduced gap between training and validation metrics across all models indicates that 300% augmentation effectively mitigated overfitting, producing more generalizable classifiers. The loss values remained consistently low across all architectures, confirming stable convergence.

**Figure 6:**
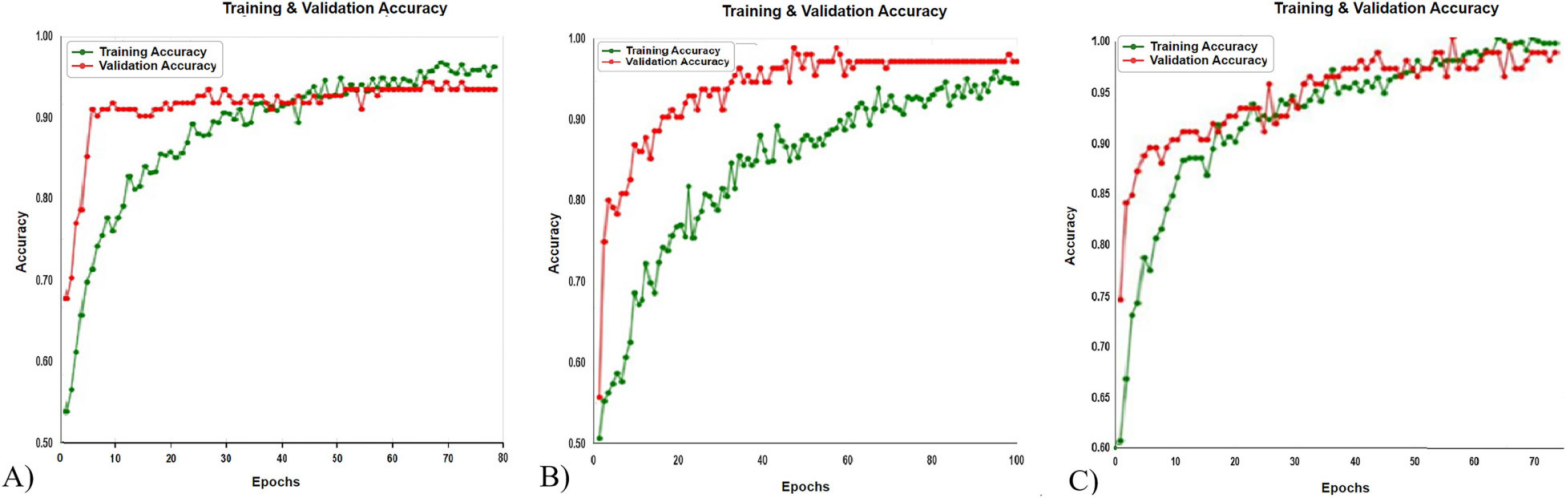
Training and validation results in Stage 3 for **(A)** ResNet50V2, **(B)** DenseNet121, **(C)** inception.

Figure 6 shows the graphs of the accuracy and loss curve during training and validation of Stage 3. The green line corresponds to the precision and the red line to the loss value of the (A) ResNet50V2, (B) DenseNet121, (C) Inception model in Stage 3.

### 4.2 Evaluation results on the test set

The trained models were evaluated on the held-out test set (30%) using accuracy, precision, recall, and *F*1-score as performance metrics.

#### 4.2.1 Stage 1: without data augmentation

**Figure 7:**
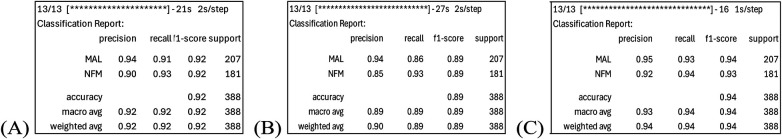
Stage 1 on testing: **(A)** ResNet50V2, **(B)** DenseNet121, **(C)** InceptionV3.

Figure 7 presents the classification reports for each architecture on the test process in Stage 1.

InceptionV3 achieved the highest test accuracy (93%) with weighted precision, recall, and *F*1-score of 0.94, demonstrating a well-calibrated classifier across both diagnostic categories. ResNet50V2 obtained 92% accuracy with balanced metrics of 0.92, while DenseNet121 achieved 89% accuracy with weighted metrics of 0.89, indicating adequate but comparatively lower classification performance.

#### 4.2.2 Stage 2: generation of new images at 50%

**Figure 8:**
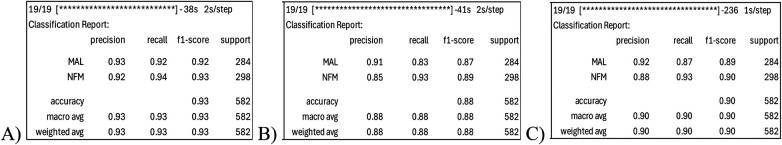
Stage 2 on testing: **(A)** ResNet50V2, **(B)** DenseNet121 and **(C)** InceptionV3.

Figure 8 presents the classification reports for each architecture on the test process in Stage 2.

ResNet50V2 achieved the highest accuracy (93%) with weighted metrics of 0.93, confirming its strong ability to distinguish between MAL and NFM categories. InceptionV3 obtained 90% accuracy with balanced metrics of 0.90, while DenseNet121 reached 88% accuracy with weighted metrics of 0.88. These results indicate that moderate augmentation benefited ResNet50V2 the most, while DenseNet121 and InceptionV3 showed marginal changes relative to Stage 1.

#### 4.2.3 Stage 3: generation of new images at 300%

**Figure 9:**
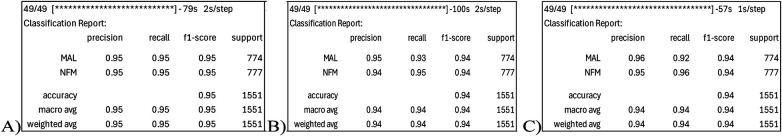
Stage 3 on testing: **(A)** ResNet50V2, **(B)** DenseNet121 and **(C)** InceptionV3.

Figure 9 presents the classification reports for each architecture on the test process in Stage 3.

With 300% augmentation, all architectures achieved their highest test accuracies. ResNet50V2 reached 95% accuracy with weighted precision, recall, and *F*1-score of 0.95, demonstrating the most consistent improvement across augmentation stages. Both DenseNet121 and InceptionV3 achieved 94% accuracy with weighted metrics of 0.94, confirming that substantial data augmentation improves generalization performance across architectures. These results are consistent with findings in the literature indicating that augmentation strategies are particularly beneficial when working with limited medical imaging datasets (32).

### 4.3 Ensembles voting classifier results

The ensemble voting classifier was applied to combine the predictions of the individual models, evaluating all possible pairwise and three-model combinations using both hard and soft voting strategies across the three augmentation stages.

#### 4.3.1 Stage 1: without data augmentation

Table 10 presents the ensemble results for Stage 1. The ResNet + Inception combination with soft voting achieved the highest accuracy (0.9459), outperforming all individual models and other ensemble configurations. The three-model ensemble (ResNet + DenseNet + Inception) achieved 0.9355 with hard voting strategies, which did not surpass the best pairwise combination.

**Table 10:** Ensemble evaluation metrics (Stage 1).

Models	Accuracy	Voting
ResNet + Inception	0.9252	Hard
ResNet + Inception	0.94587	Soft
DenseNet + Inception	0.9226	Hard
DenseNet + Inception	0.9355	Soft
ResNet + DenseNet	0.91237	Hard
ResNet + DenseNet	0.91752	Soft
*ResNet + DenseNet + Inception*	*0* *.* *9355*	*Hard*
ResNet + DenseNet + Inception	0.9355	Soft

#### 4.3.2 Stage 2: generation of new images at 50%

Table 11 presents the ensemble results for Stage 2. The ResNet + DenseNet + Inception combination with hard voting achieved the highest accuracy (0.9243), followed closely by ResNet + DenseNet with soft voting (0.9226) and ResNet + Inception with soft voting (0.9226). The marginal differences among the top-performing ensembles suggest that the three architectures provide complementary predictions at this augmentation level.

**Table 11:** Ensemble evaluation metrics (Stage 2).

Models	Accuracy	Voting
ResNet + Inception	0.9209	Hard
ResNet + Inception	0.9226	Soft
DenseNet + Inception	0.8986	Hard
DenseNet + Inception	0.914	Soft
ResNet + DenseNet	0.9106	Hard
ResNet + DenseNet	0.9226	Soft
*ResNet + DenseNet + Inception*	*0* *.* *9243*	*Hard*
ResNet + DenseNet + Inception	0.9192	Soft

#### 4.3.3 Stage 3: generation of new images at 300%

Table 12 presents the ensemble results for Stage 3. The ResNet + DenseNet + Inception ensemble with soft voting achieved the highest overall accuracy across all stages (0.9658), followed by ResNet + DenseNet + Inception with hard voting (0.9638). Notably, this was the only stage in which the three-model ensemble consistently outperformed all pairwise combinations, indicating that with sufficient training data, the complementary features captured by the three architectures can be effectively leveraged through ensemble aggregation. The soft voting strategy outperformed hard voting in most configurations, which aligns with evidence that probability-based aggregation preserves richer decision information than majority-class selection (28).

**Table 12:** Ensemble evaluation metrics (Stage 3).

Models	Accuracy	Voting
ResNet + Inception	0.9587	Hard
ResNet + Inception	0.9516	Soft
DenseNet + Inception	0.9509	Hard
DenseNet + Inception	0.9638	Soft
ResNet + DenseNet	0.9464	Hard
ResNet + DenseNet	0.9626	Soft
ResNet + DenseNet + Inception	0.9638	Hard
*ResNet + DenseNet + Inception*	*0* *.* *9658*	*Soft*

### 4.4 Statistical significance analysis

To assess whether the observed differences in classification accuracy between models are statistically significant, a *Z*-test for two proportions was applied to all key pairwise comparisons. This test evaluates whether two observed proportions (accuracies) differ significantly given the sample sizes, under the null hypothesis that both models perform equally. Table 13 presents the results for the local dataset (*n* = 388) and the external Kaggle dataset (*n* = 693).

**Table 13:** *Z*-test for two proportions: pairwise model comparisons.

Comparison of Model	Dataset	Acc. Model 1	Acc. Model 2	*Δ* (pp)	*Z*	*p*-value	Sig.
ResNet + DenseNet (soft) vs. ResNet50V2	Local (*n* = 388)	96.26%	95.00%	1.26	0.86	0.391	ns
ResNet + DenseNet (soft) vs. DenseNet121	Local (*n* = 388)	96.26%	94.00%	2.26	1.46	0.145	ns
ResNet + DenseNet (soft) vs. InceptionV3	Local (*n* = 388)	96.26%	94.00%	2.26	1.46	0.145	ns
ResNet + DenseNet + Inception (soft) vs. ResNet50V2	Local (*n* = 388)	96.58%	95.00%	1.58	1.11	0.268	ns
ResNet + DenseNet (soft) vs. ResNet50V2	External (*n* = 693)	89.61%	80.37%	9.24	4.82	<0.001	***
ResNet + DenseNet (soft) vs. InceptionV3	External (*n* = 693)	89.61%	72.72%	16.89	8.04	<0.001	***
ResNet + DenseNet (soft) vs. ResNet + DenseNet + Inception (soft)	External (*n* = 693)	89.61%	84.41%	5.2	2.88	0.004	**

ns, not significant (*p* ≥ 0.05).

** *p* < 0.01.

*** *p* < 0.001.

On the local dataset, none of the pairwise differences between the ensemble and individual models reached statistical significance at the *α* = 0.05 level. This is attributable to the relatively small test set (*n* = 388) and the high baseline accuracy of all models (94%–96%), which reduces statistical power to detect small effect sizes. However, on the larger external Kaggle dataset (*n* = 693), the differences between the ResNet + DenseNet ensemble and the individual models were highly significant: compared to ResNet50V2 (*Z* = 4.816, *p* < 0.001) and InceptionV3 (*Z* = 8.042, *p* < 0.001). Furthermore, the ResNet + DenseNet pairwise ensemble significantly outperformed the three-model ensemble on external data (*Z* = 2.879, *p* = 0.004), providing statistical confirmation that the inclusion of InceptionV3 degrades cross-domain performance.

Additionally, the accuracy drop between local and external validation for the same model was statistically significant: the ResNet + DenseNet ensemble achieved 96.26% locally vs. 89.61% externally (*Z* = 3.855, *p* < 0.001), confirming that the observed domain shift represents a genuine reduction in performance rather than sampling variability.

Overall, the results demonstrate a consistent improvement in classification performance as data augmentation increases, with the ensemble approach yielding the highest accuracies in all three stages. The best individual model (ResNet50V2 at 95% in Stage 3) was surpassed by all top ensemble configurations, confirming the added value of ensemble strategies for pleural fluid cytology classification. On the local dataset, the highest accuracy was achieved by ResNet + DenseNet + Inception with soft voting (96.58%), followed closely by ResNet + DenseNet with soft voting (96.26%). However, as will be demonstrated in Section 5 through external validation, the ResNet + DenseNet ensemble with soft voting is ultimately selected as the optimal model due to its superior cross-dataset generalizability—a criterion that is more clinically relevant than marginal gains on the local test set alone.

## 5 Validation in external datasets

To assess the generalizability of the trained models beyond the local clinical dataset, an external validation was performed using the Body Cavity Fluid Cytology Images repository available on Kaggle (34). This dataset comprises 693 images, 533 classified as malignant (MAL) and 160 as negative for malignancy (NFM), acquired at 40× magnification and resized to 250 × 250 pixels to match the input dimensions of the trained models. The class distribution is presented in Table 14. It is important to note that this dataset exhibits a substantial class imbalance (77% MAL vs. 23% NFM), in contrast to the nearly balanced local dataset, which constitutes a more demanding test of model robustness.

**Table 14:** Distribution of images in the Kaggle dataset.

Category	Description	Images
MAL	Malignant	533
NFM	Negative for malignancy	160
TOTAL	Total	693

### 5.1 Individual model evaluation

Table 15 presents the evaluation results of the three base architectures across the three augmentation stages on the Kaggle dataset. DenseNet121 consistently achieved the highest accuracy across all stages, reaching its peak (0.8961) in Stage 3 with 300% augmentation. ResNet50V2 improved from Stage 1 (0.72) to Stage 2 (0.8196) but slightly decreased in Stage 3 (0.8037), suggesting that excessive augmentation may introduce noise that degrades its feature representations on external data. InceptionV3 exhibited the lowest performance overall, with a notable decline in Stage 3 (0.7272), indicating that this architecture is more sensitive to domain shift when combined with aggressive augmentation. These results suggest that model architecture plays a more decisive role than augmentation volume in cross-dataset classification performance, with DenseNet121 proving to be the most robust individual classifier, likely due to its dense connectivity pattern, which promotes feature reuse and reduces overfitting to domain-specific patterns.

**Table 15:** Individual model evaluation by stage on the Kaggle dataset.

Stage	Base architecture	Accuracy
Stage 1: Without data augmentation	ResNet50V2	0.72
	DenseNet121	0.89
	InceptionV3	0.76
Stage 2: Generation at 50%	ResNet50V2	0.8196
	DenseNet121	0.8744
	InceptionV3	0.7676
Stage 3: Generation at 300%	ResNet50V2	0.8037
	DenseNet121	0.8961
	InceptionV3	0.7272

### 5.2 Ensemble voting classifier evaluation

Table 16 presents the ensemble evaluation results across the three augmentation stages on the Kaggle dataset. Four architecture combinations: ResNet + Inception, DenseNet + Inception, ResNet + DenseNet, and ResNet + DenseNet + Inception, were evaluated using both hard and soft voting strategies.

**Table 16:** Ensemble evaluation by stage on the Kaggle dataset.

Stage	Base architecture	Accuracy	Voting classifier
Stage 1: Without data augmentation	ResNet + Inception	0.8196	Hard
	ResNet + Inception	0.7662	Soft
	DenseNet + Inception	0.8816	Hard
	DenseNet + Inception	0.8759	Soft
	*ResNet + DenseNet*	*0* *.* *8917*	*Hard*
	ResNet + DenseNet	0.8629	Soft
	ResNet + DenseNet + Inception	0.82395	Hard
	ResNet + DenseNet + Inception	0.8412	Soft
Stage 2: Generation at 50%	ResNet + Inception	0.8412	Hard
	ResNet + Inception	0.8167	Soft
	DenseNet + Inception	0.8643	Hard
	DenseNet + Inception	0.8787	Soft
	*ResNet + DenseNet*	*0* *.* *8715*	*Hard*
	ResNet + DenseNet	0.8802	Soft
	ResNet + DenseNet + Inception	0.8542	Hard
	ResNet + DenseNet + Inception	0.8658	Soft
Stage 3: Generation at 300%	ResNet + Inception	0.8095	Hard
	ResNet + Inception	0.7705	Soft
	DenseNet + Inception	0.8672	Hard
	DenseNet + Inception	0.886	Soft
	*ResNet + DenseNet*	*0* *.* *8831*	*Hard*
	ResNet + DenseNet	0.8961	Soft
	ResNet + DenseNet + Inception	0.8311	Hard
	ResNet + DenseNet + Inception	0.8441	Soft

In Stage 1, the ResNet + DenseNet combination with hard voting achieved the highest accuracy (0.8917), followed by DenseNet + Inception with soft voting (0.8759). In Stage 2, the ResNet + DenseNet ensemble with soft voting reached the highest accuracy (0.8802), with hard voting achieving 0.8715. In Stage 3, the ResNet + DenseNet combination with soft voting attained the highest overall accuracy across all stages and configurations on the external dataset (0.8961), matching the individual DenseNet121 performance, while hard voting achieved 0.8831.

These external validation results are critical for model selection. Although the ResNet + DenseNet + Inception ensemble with soft voting achieved the highest accuracy on the local dataset (96.58%), it did not maintain this advantage on external data, reaching only 84.41% in Stage 3. In contrast, the ResNet + DenseNet ensemble with soft voting achieved 96.26% on the local dataset, only 0.32 percentage points below the three-model ensemble, while attaining 89.61% on the external dataset, representing a 5.20 percentage point advantage over the three-model ensemble on unseen data. This trade-off between local optimality and cross-domain robustness justifies the selection of ResNet + DenseNet with soft voting as the optimal model for clinical deployment, where performance on diverse, unseen samples is more important than marginal improvements on a single test set.

Two consistent patterns emerge from these results. First, the ResNet + DenseNet combination consistently outperformed all other ensemble configurations across every stage, confirming the complementarity between ResNet50V2's residual learning and DenseNet121's dense connectivity for pleural cytology classification. Second, the three-model ensemble (ResNet + DenseNet + Inception) did not yield the best results in any stage, suggesting that InceptionV3's lower individual performance on the external dataset introduces conflicting predictions that dilute the ensemble's effectiveness rather than enhancing it. This finding aligns with evidence that ensemble performance depends not only on the diversity of component models but also on their individual competence—weaker classifiers can degrade aggregate performance when their error patterns are not sufficiently orthogonal to those of stronger models (35).

Comparing the local and external validation results, the models exhibited the expected drop in accuracy when evaluated on the Kaggle external dataset. The best-performing ensemble on the local test set ResNet + DenseNet + Inception with soft voting achieved 96.58%, whereas the top ensemble on the external dataset ResNet + DenseNet with soft voting reached 89.61%. This decrease can be attributed to domain shift, likely caused by differences in staining protocols, imaging equipment, magnification levels, and class distribution between the two datasets. Despite this reduction, the best ensemble maintained an accuracy close to 90%, indicating acceptable cross-domain generalizability for a clinical decision-support tool.

## 6 Discussion

This study proposed an ensemble deep learning framework based on transfer learning for the binary classification of pleural fluid cytology images into malignant (MAL) and negative for malignancy (NFM) categories. The discussion examines the obtained results in relation to prior work, analyzes the contribution of ensemble strategies and data augmentation, and addresses the limitations and generalizability of the proposed approach.

### 6.1 Performance of individual models in context

The best individual model on the local dataset was ResNet50V2, which achieved 95% test accuracy in Stage 3 (300% augmentation), followed by DenseNet121 and InceptionV3, both at 94%. These results are comparable to those reported in the literature for cytological image classification. A deep CNN applied to 404 cytological pleural effusion cases achieved 91.67% accuracy, 87.50% sensitivity, and 94.44% specificity while a U-Net architecture for malignant cell detection in pleural fluid reported precision, recall, and specificity values of 0.96, 0.96, and 0.97, respectively (21). The individual accuracies obtained in the present study (94%–95%) exceed the accuracy reported by (14), although direct comparison is limited by differences in dataset composition, image resolution, and evaluation protocols, notably, this study used whole-slide images from a single institution with 60 test cases, whereas the present work evaluated on a fixed test set of 388 unaugmented images across all stages, providing a consistent and unbiased evaluation base.

In the external validation on the Kaggle dataset, DenseNet121 achieved the highest individual accuracy (0.8961 in Stage 3), outperforming ResNet50V2 (0.8037) and InceptionV3 (0.7272). The reversal in architecture ranking between the local and external datasets, where ResNet50V2 led on local data but DenseNet121 dominated on external data, suggests that DenseNet121's dense connectivity pattern (see Section 3.3) confers greater robustness to domain shift arising from differences in staining protocols and imaging conditions between the two datasets (33). This observation aligns with findings demonstrating that DenseNet architectures exhibit stronger generalization in transfer learning scenarios with limited or heterogeneous medical imaging data (25).

### 6.2 Added value of ensemble voting strategies

The ensemble voting classifier consistently outperformed individual models across all augmentation stages. On the local dataset, the best ensemble ResNet + DenseNet + Inception with soft voting, achieved 96.58% accuracy in Stage 3, surpassing the best individual model (ResNet50V2 at 95%) by 1.58 percentage points.

The *Z*-test for two proportions revealed that the accuracy differences between ensemble and individual models on the local dataset did not reach statistical significance (*p* > 0.05 for all comparisons), which is consistent with the limited statistical power afforded by a test set of 388 images when baseline accuracies exceed 94%. Nevertheless, on the external dataset (*n* = 693), the ResNet + DenseNet ensemble with soft voting significantly outperformed both ResNet50V2 (*Z* = 4.816, *p* < 0.001) and InceptionV3 (*Z* = 8.042, *p* < 0.001), demonstrating that the ensemble's advantage becomes statistically robust when evaluated under more challenging cross-domain conditions. This pattern suggests that the true value of the ensemble approach lies not in marginal accuracy gains on well-matched local data, but in its statistically significant superior generalizability to unseen data from independent sources.

This improvement, while modest in absolute terms, is clinically meaningful in a diagnostic support context where incremental gains in accuracy reduce the rate of missed malignancies.

These findings are consistent with the ensemble learning literature in medical imaging. Hybrid models combining ResNet-50 deep features with an ensemble of five classifiers achieved 98.00% and 99.32% accuracy on histological image datasets (25), confirming that ensemble strategies effectively exploit complementary features from different architectures. Similarly, deep ensemble learning integrating sparse regression models demonstrated improved accuracy and generalization for brain disease diagnosis compared to individual models (28). The present study extends these principles specifically to pleural effusion cytology, a domain where ensemble approaches had not been previously explored.

On the external Kaggle dataset, the ResNet + DenseNet combination with soft voting achieved the highest ensemble accuracy (0.8961 in Stage 3), consistently outperforming all other configurations across every stage. Notably, the three-model ensemble (ResNet + DenseNet + Inception) did not yield the best external results in any stage, suggesting that InceptionV3's weaker cross-domain performance introduces conflicting predictions that dilute the ensemble's effectiveness. This observation is statistically supported by the *Z*-test for two proportions, which confirmed that the ResNet + DenseNet pairwise ensemble significantly outperformed the ResNet + DenseNet + Inception three-model ensemble on the external dataset (*Z* = 2.879, *p* = 0.004). This aligns with evidence that ensemble performance depends not only on model diversity but also on the individual competence of component classifiers weaker models can degrade aggregate performance when their error patterns are not sufficiently complementary (35).

### 6.3 Impact of data augmentation

A progressive improvement in classification performance was observed as data augmentation increased from Stage 1 (no augmentation) through Stage 3 (300% augmentation). On the local dataset, the best individual accuracy improved from 93% (InceptionV3, Stage 1) to 95% (ResNet50V2, Stage 3), and the best ensemble accuracy improved from 94.59% (Stage 1) to 96.58% (Stage 3). These results confirm that geometric augmentation strategies, including flips, rotations, translations, and zoom, effectively increase the diversity of the training distribution and reduce overfitting, consistent with findings in the augmentation literature for deep learning (32).

However, the external validation results revealed a more nuanced picture. While DenseNet121 benefited consistently from augmentation (accuracy improved from 0.89 in Stage 1 to 0.8961 in Stage 3), InceptionV3 exhibited a decline from 0.76 (Stage 1) to 0.7272 (Stage 3), and ResNet50V2 peaked at Stage 2 (0.8196) before decreasing at Stage 3 (0.8037). These divergent responses suggest that aggressive augmentation may amplify domain-specific artifacts that hinder generalization for certain architectures, particularly when the external dataset differs substantially in class distribution (77% MAL vs. 23% NFM in Kaggle compared to the balanced local dataset) and imaging conditions.

### 6.4 Comparison with state-of-the-art approaches

Based on the results presented in Sections 4 and 5, the ResNet + DenseNet ensemble with soft voting is selected as the optimal model for pleural fluid cytology classification. Although the ResNet + DenseNet + Inception ensemble with soft voting achieved the highest local accuracy (96.58%), the ResNet + DenseNet configuration with soft voting (96.26% local, 89.61% external) demonstrated the most robust cross-dataset generalization, outperforming all other ensemble configurations on the external Kaggle dataset across every augmentation stage. This selection prioritizes generalizability over marginal local gains—a criterion that is more relevant for clinical deployment where the model must perform reliably on samples from diverse institutional settings.

The proposed ensemble framework addresses a gap identified in the literature: while prior studies have applied individual deep learning models to pleural effusion cytology (14, 21) or multi-scale interpretable architectures (22), none had systematically combined multiple pre-trained architectures with ensemble voting for this specific task. To contextualize the performance of the proposed model within the broader landscape of cytological and histopathological image classification, Table 17 presents a consolidated comparison of performance metrics reported in related studies alongside the results obtained in this work.

**Table 17:** Comparison of performance metrics between prior studies and the proposed model.

Study	Application domain	Key metric	Value
Aboobacker et al. (21)	Pleural fluid cytology	Precision; Recall; Specificity; AUC-ROC	0.96; 0.96; 0.97; 0.97
Xie et al. (14)	Pleural effusion cytology	Accuracy; Sensitivity; Specificity; AUC-ROC	91.67%;87.50%; 94.44%; 0.9526
Yu et al. (20)	Cerebrospinal fluid cytology	Accuracy (cell type); mAP (subtype)	96.15%; 78%
Carlo Bruno et al. (23)	General cytology (anomaly detection)	Recall; Precision; AUROC	1.00; 98.39%; 99.98%
de Oliveira et al. (25)	Histological image classification	Accuracy (best hybrid model)	98.00%; 99.32%
Slabaugh et al. (11)	Thyroid cytology and histopathology (review)	FNA sensitivity; specificity (range)	68%–98%; 56%–100%
Proposed model (this study)	Pleural fluid cytology	Accuracy	96.26%

The proposed ResNet + DenseNet ensemble with soft voting (96.26% accuracy) surpasses the most directly comparable study in pleural effusion cytology by Xie et al., (14), who reported 91.67% accuracy using a deep CNN on 60 test whole-slide images. The improvement of 4.59 percentage points is noteworthy given that the present study was evaluated on a test set of 388 unaugmented images, substantially larger than the 60 WSIs used by (14) providing a more statistically robust estimate of classification performance. Additionally, the proposed model slightly exceeds the 96.15% accuracy reported by Yu et al. (20), for cell-type classification in cerebrospinal fluid cytology, although this comparison is limited by differences in body fluid type and classification complexity.

Regarding studies that report higher performance metrics, the weakly supervised PatchCore approach achieved an AUROC of 99.98% on cytology images (23); however, this method employs anomaly detection using only image-level annotations, which represents a fundamentally different evaluation paradigm from supervised binary classification. Similarly, hybrid models combining ResNet-50 features with ensemble classifiers achieved 98.00% and 99.32% accuracy on histological image datasets (25), but histological preparations typically exhibit more homogeneous and well-defined morphological patterns than pleural fluid cytology, where cell morphology is affected by fluid processing, staining variability, and the presence of reactive mesothelial cells that can mimic malignant cells.

The interpretable multi-scale attention framework (IMA-SSL) proposed by Wang et al. (22), outperformed five state-of-the-art models on 1,250 whole-slide images of pleural effusion; however, that approach relies on a single sophisticated architecture with self-supervised pre-training, whereas the present work demonstrates that competitive performance can be achieved through the strategic combination of simpler, widely available pre-trained models, an approach that is more accessible for implementation in resource-constrained clinical settings. In the broader context of systematic reviews, the proposed model's accuracy falls within the upper performance range reported for both non-gynecological cancer cytopathology (13), and thyroid FNA cytology with sensitivity of 68%–98% and specificity of 56%–100% (11), despite addressing a more complex cytological domain where inter-observer variability and morphological ambiguity remain significant diagnostic challenges.

Collectively, these comparisons confirm that the proposed ensemble framework achieves state-of-the-art performance for pleural fluid cytology classification while maintaining practical advantages in terms of implementation simplicity and reproducibility.

## 7 Conclusions

This study designed, implemented, and evaluated an ensemble deep learning framework based on transfer learning for the automatic classification of pleural fluid cytology images into malignant (MAL) and negative for malignancy (NFM) categories, following the criteria of the International System for Reporting Serous Fluid Cytopathology (TIS). Three pre-trained convolutional neural network architectures: ResNet50V2, DenseNet121, and InceptionV3, were systematically combined through hard and soft voting strategies across three data augmentation stages, and validated on both a local clinical dataset from Hospital Nacional Cayetano Heredia comprising 1,292 annotated images and an external public dataset from Kaggle comprising 693 images.

The ensemble approach demonstrated a consistent and measurable advantage over individual models across all experimental conditions. On the local dataset, the ResNet + DenseNet + Inception ensemble with soft voting achieved the highest accuracy (96.58%), followed by ResNet + DenseNet with soft voting (96.26%). However, when considering cross-dataset generalizability, evaluated on an independent external dataset, the ResNet + DenseNet ensemble with soft voting was selected as the optimal model, achieving 96.26% on the local test set and 89.61% on the external dataset, surpassing the best individual model (ResNet50V2 at 95%) by 1.26 percentage points on local data while maintaining the strongest performance on unseen external data. This result confirms that combining architectures with complementary feature extraction mechanisms (as described in Section 3.3), effectively captures a broader range of discriminative cytological features than any single architecture alone, producing a more robust and reliable classifier for the binary distinction between malignant and non-malignant pleural fluid specimens. The soft voting strategy, which averages predicted probabilities across models before selecting the final class, consistently outperformed hard voting in the majority of configurations, indicating that preserving probabilistic information during aggregation yields more nuanced and accurate classification decisions than simple majority-class selection.

Data augmentation proved essential for improving classification performance on the local dataset. The best individual accuracy improved from 93% without augmentation to 95% with 300% augmentation, and the best ensemble accuracy increased from 94.59% to 96.58% across the same stages. The geometric transformations applied, including horizontal and vertical flips, rotations up to 30 degrees, translations of up to 15%, and zoom variations of 15%, effectively expanded the diversity of the training distribution and reduced overfitting, enabling the models to learn features that are invariant to position, orientation, and scale. However, the external validation revealed that the benefit of augmentation is architecture-dependent: DenseNet121 improved consistently across all stages, whereas InceptionV3 exhibited a notable decline in accuracy from 76% to 72.72% with aggressive augmentation, and ResNet50V2 peaked at 50% augmentation before decreasing at 300%. This finding underscores the importance of evaluating augmentation strategies on external data rather than relying solely on internal validation metrics to draw conclusions about model generalizability.

Cross-dataset generalizability was confirmed through external validation on the Kaggle Body Cavity Fluid Cytology dataset. The ResNet + DenseNet ensemble with soft voting achieved 89.61% accuracy on this external dataset, the highest among all tested configurations, despite substantial differences in class distribution (77% malignant vs. 23% negative for malignancy, compared to the nearly balanced local dataset), staining protocols, magnification levels, and imaging equipment. The accuracy reduction of approximately 6.65 percentage points relative to the local dataset is consistent with the expected effects of domain shift in medical imaging and confirms that the model maintains clinically acceptable performance when applied to unseen data from an independent source with different acquisition conditions.

The inclusion of InceptionV3 in the three-model ensemble did not improve performance compared to the pairwise ResNet + DenseNet combination on either the local or external dataset. This demonstrates that ensemble effectiveness depends not only on model diversity but critically on the individual competence of each component classifier. Incorporating a weaker model can introduce conflicting or unreliable predictions that dilute the ensemble's discriminative capacity rather than enhance it, highlighting the need for careful model selection prior to ensemble construction.

The proposed system constitutes a decision-support tool for pathologists rather than a fully autonomous diagnostic system. Its clinical value lies in providing a preliminary classification that could prioritize specimens requiring urgent pathological review, reduce the time interval between sample processing and diagnostic reporting, and mitigate the inter-observer variability that remains a persistent challenge in cytological evaluation. The reliance on standard pre-trained architectures available through open-source frameworks and a straightforward ensemble voting mechanism makes the approach fully reproducible and deployable without requiring specialized hardware, proprietary models, or self-supervised pre-training pipelines. These characteristics are especially relevant for healthcare systems in developing countries where access to specialized pathologists is limited and computational resources may be constrained, positioning the proposed framework as a practical and scalable solution for computer-aided diagnosis in pleural fluid cytopathology.

## 8 Limitations and future work

This study has some limitations that provide opportunities for further improvement. First, the local dataset consists of 1,292 images from a single institution, which, although adequate for the proposed binary classification task, could be expanded to better reflect broader clinical variability. Similarly, external validation was conducted using one publicly available dataset with an imbalanced class distribution, which may not fully represent diverse real-world scenarios. Second, the study focuses on binary classification (malignant vs. negative for malignancy), which is appropriate for initial diagnostic support but does not yet address detailed subtyping of malignancies. Third, the use of grayscale images helped reduce staining variability, although it may limit the exploitation of potentially useful color information. Additionally, the models were evaluated retrospectively on curated datasets and not yet assessed in prospective clinical workflows. Fourth, the present study did not compute AUC-ROC curves for the proposed models, relying instead on accuracy, precision, recall, and *F*1-score as evaluation metrics. Fifth, the *Z*-test for two proportions was applied to assess pairwise accuracy differences, this test treats the predictions of each model as independent samples, which is an approximation given that both models are evaluated on the same test set. More rigorous paired tests such as McNemar's test, which accounts for the dependency structure of paired predictions, could not be applied because individual per-sample prediction vectors were not retained. Finally, rare malignant subtypes are underrepresented, which may influence performance in less common cases.

Future work can build upon these results in several directions. Expanding the dataset through multi-institutional collaborations would enhance generalizability and robustness. Extending the framework to multi-class classification could provide more detailed diagnostic support. The incorporation of interpretability techniques, such as visual explanation methods, may further improve clinical trust and usability. Moreover, exploring advanced stain normalization approaches could allow the model to leverage both color and structural information while maintaining robustness. Integration with whole-slide imaging pipelines represents another promising direction for scaling the system to real clinical environments. Finally, prospective validation studies would help assess the system's performance in routine practice and support its potential adoption as a clinical decision-support tool.

## Data Availability

The original contributions presented in the study are included in the article/Supplementary Material, further inquiries can be directed to the corresponding author.
